# Functionalization of Polyvinyl pyrrolidone/Polyvinyl alcohol blends doped with zinc sulfate and irradiated with electron beam

**DOI:** 10.1038/s41598-026-45515-z

**Published:** 2026-04-13

**Authors:** Heba Abdelmaksoud, M. Salah, Khaled M. Zakaria, F. A. Abdel-Wahab

**Affiliations:** 1 Physics Department, Faculty of Science, Ain Shams University, Cairo, 11566 Egypt; 2 Polymer Chemistry Department, National Center for Radiation Research and Technology, Egyptian Atomic Energy Authority, Cairo, Egypt; 3 Radiation Safety Department, Nuclear and Radiological Safety Research Centre (NRSRC), Egyptian Atomic Energy Authority (EAEA), Cairo, Egypt

**Keywords:** PVA/PVP blend, Microstructure, Thermal stability, AC conductivity, Dielectric relaxation, Chemistry, Materials science, Nanoscience and technology, Physics

## Abstract

Nanocomposite films of poly(vinyl alcohol) (PVA) and poly(vinylpyrrolidone) (PVP) doped with Zn^2+^ ions are prepared using in situ chemical method. The as-prepared samples are irradiated with an electron beam at doses of 20, 25, 30, 35, and 40 kGy. As demonstrated by the measured X-ray diffraction spectra, the films exhibited a polycrystalline nature with substantial enhancement of crystallinity from 6.2% to 27.5% with increasing irradiation dose. This finding is indicative of enhanced polymer chain ordering. Field emission scanning electron microscopy revealed uniform dispersion of Zn^2+^ nanoparticles accompanied by an increase in particle size from 3.58 to 4.25 μm against applied radiation doses. The measured infrared spectra demonstrated an interaction between hydroxyl group in PVA and Carbonyl group in PVP accompanied by an increase in the number of free ions with increasing irradiation dose. Thermal spectrum measurements conducted using differential thermal analysis and thermogravimetric techniques demonstrated an enhancement in chemical stability as evidenced by an increase in glass transition temperature from 33.2 °C to 35.6 °C. Kinetic spectrum analysis using Coats–Redfern method revealed an increase in activation energy, enthalpy, and Gibbs free energy against irradiation dose. The impedance and dielectric measurements exhibited non-Debye relaxation, with Nyquist plots showing reduced semicircle diameters at elevated temperatures. The highest dielectric constant was observed at a dose of 40 kGy. This find establishes a correlation between irradiation induced crystallinity and nanoparticle polymer interaction and demonstrated an enhancement in electrical performance.

## Introduction

The hydroxyl groups present within the carbon backbone of PVA are of pivotal significance to the functionality of the material. The formation of hydrogen bonds is a consequence of these groups, and these bonds contribute to the polymer’s mechanical strength, film-forming ability, and water solubility^1,2^. The hydrogen bonds thus formed enhance the interaction of PVA with other materials, rendering it suitable for blending with other polymers, forming composites, and acting as a matrix in various applications, including hydrogels, adhesives, and packaging materials. Its biocompatibility and non-toxicity broaden its use in biomedical applications, such as systems for delivering medications, wound dressings, and tissue engineering^3,4^. Furthermore, when PVA is thermally heated at temperatures above 300 °C, it undergoes a chemical rearrangement process that promotes the formation of hydrogen bonds with water molecules. The thermal treatment under investigation has been shown to induce modifications that not only serve to strengthen the material, but also result in the generation of highly conjugated aromatic structures.

Polyvinyl pyrrolidone (PVP) is a synthetic polymer known for its unique properties, primarily due to the presence of the pyrrolidone ring in its structure. The lactam ring in PVP contains a peptide bond, which gives the polymer its planar and highly polar characteristics^5^. This polarity makes PVP highly hydrophilic and capable of forming strong interactions with water and other polar substances. The planar structure of the pyrrolidone ring allows for efficient stacking and interaction with other molecules, which explains why PVP is widely used as a stabilizer, binder, and dispersing agent in various applications^6^. Furthermore, Polyvinyl pyrrolidone (PVP) is indeed an amorphous polymer, which means it lacks a crystalline structure and has a disordered arrangement of its molecular chains^7^. Presence of the rigid pyrrolidone group in PVP contributes significantly to its high glass transition temperature (*T*_g_) which is argued to the restricted mobility of the polymer chains, and due to the stiff and planar nature of the pyrrolidone ring^8^. Upon thermal decomposition, PVP experiences bond breaking in its polymer backbone and the removal of side groups, leading to thermally induced chain scissions. This process yields various decomposition products including ester groups, ammonia (due to protonation of NH_2_ molecules), hydrocarbons, and pyrrolidone molecules^9^.

When PVA and PVP are combined, hydrogen bonds can form between the hydroxyl groups of PVA and the carbonyl groups of the pyrrolidone ring in PVP^8,9^. These interactions enhance the miscibility of the two polymers and result in the formation of a stable blend. Moreover, the blend of PVA/PVP leads to physical cross-links within the hydrogel network^10,11^. These cross-links provide structural stability, which is particularly important for maintaining the integrity of the hydrogel in physiological environments. As a result, these hybrid materials emerge as multifunctional advanced polymers offering vast potential for technological applications, including the fabrication of next-generation optoelectronic devices, solar cells, energy storage systems, and nanodielectric applications^12–14^.

Zinc sulfate heptahydrate (ZnSO_4_·7H_2_O) is a compound composed of zinc, sulfur, and oxygen. When transformed into nanoparticles, it shows distinct properties, including high electrical conductivity, remarkable plasmonic activity, catalytic efficiency, and potent antimicrobial effects^15,16^. These nanoparticles can be further encapsulated or dispersed within a polymer matrix, which is commonly composed of polyvinyl alcohol (PVA) and polyvinyl pyrrolidone (PVP)^15–17^. Choudhary et al.^18^ reported that incorporating 1.0 wt% of ZnO into a PVA–PVP film significantly improved its performance, resulting in novel surface morphology, enhanced topography, and increased thermal stability. Zyoud et al.^19^ observed that ZnO-doped PVA/PVP polymeric films exhibited enhanced linear and nonlinear optical properties^20,21^. Doping or blending polymers with appropriate materials can substantially modify their functionality. For example, Ramesan et al.^22^ identified a blend of PVA/PVP filled with Ag-doped ZnO nanoparticles as a promising film for optoelectronic applications. Additionally, Merlin et al.^23^ used a polyaniline with ZnS nanoparticles (PANI/ZnS) to develop an optical limiting and structural parameters. Furthermore, Rani et al.^24^ synthesized functionalized or derived zinc nanoparticles and shown that zinc-based nano compounds can work as photocatalysts. Various studies have extensively explored the thermal degradation behaviour of polymeric composites and blends. For example, PVP/Ag core-shell structures have been investigated using thermogravimetric (TG) spectra to assess their structural properties^25,26^. Voronova et al.^27^ demonstrated that PVA/SiO_2_ nanocomposites exhibit superior thermal properties compared to pure PVA. In these nanocomposites, main chain degradation typically results in the release of carbon dioxide and low-molecular-weight conjugated polyenes, while side-chain degradation can produce carboxylic acid.

When ionizing radiation interacts with a polymer, it causes excitation and ionization of the material molecules, leading to the breaking of original bonds, chain scission, radical formation, and cross-linking within the polymer^28,29^. The extent of scission and cross-linking depends not only on the polymer structure but also on the energy deposited per unit track length which can, in turn, modify the polymer structure and optical properties^30,31^.

The present study aims to investigate the structural variations in PVA/PVP blended nanocomposite polymer films doped with Zn^2+^ ions and irradiated with e-beam doses at 20, 25, 30, 35, and 40 kGy. The structural characterization is conducted using XRD, FESEM, and FT-IR spectroscopy, while the impact of these variations on thermal degradation is assessed through DSC and TGA measurements. This work will also examine the relationship between structural changes and the electrical and dielectric properties of polymer nanocomposite films. The findings provide valuable insights into the role of Zn^2+^ ions in improving the properties of the composite samples.

## Experimental details

### Materials and chemicals

Nanocomposite films were synthesized using Polyvinyl Alcohol (PVA) with a molecular weight of 115,000 g/mol and Polyvinyl pyrrolidone (PVP) with a molecular weight of 40.000 g/mol. and 95% purity, both obtained from Loba Chemie, India. Zinc Sulfate (ZnSO_4_·7H_2_O) with a molecular weight of 161.47 g/mol. and Glycerine (C_3_H_8_O_3_) with a molecular mass of 92.094 g/mol. and 99.5% purity were supplied by El Gomhouria Co., Egypt. In the previous research^32^, PVA/PVP copolymer were prepared in various compositions: (0:100), (20:80), (40:60), (60:40), (80:20), and (100:0). The gelation percentage was measured to determine the optimal composition. The ratio 40:60 PVA/PVP ratio was identified as the most suitable for use as a stabilizing agent or nanoreactor in nanoparticle synthesis.

The nomenclature and terminology of the prepared samples were adopted in accordance with the recommendations of the International Union of Pure and Applied Chemistry (IUPAC). Poly(vinyl alcohol) (PVA) and Poly(vinylpyrrolidone) (PVP) were designated using systematic polymer naming conventions, and the prepared materials were classified.

### Preparation of gel electrolyte polymer

**Scheme 1:**
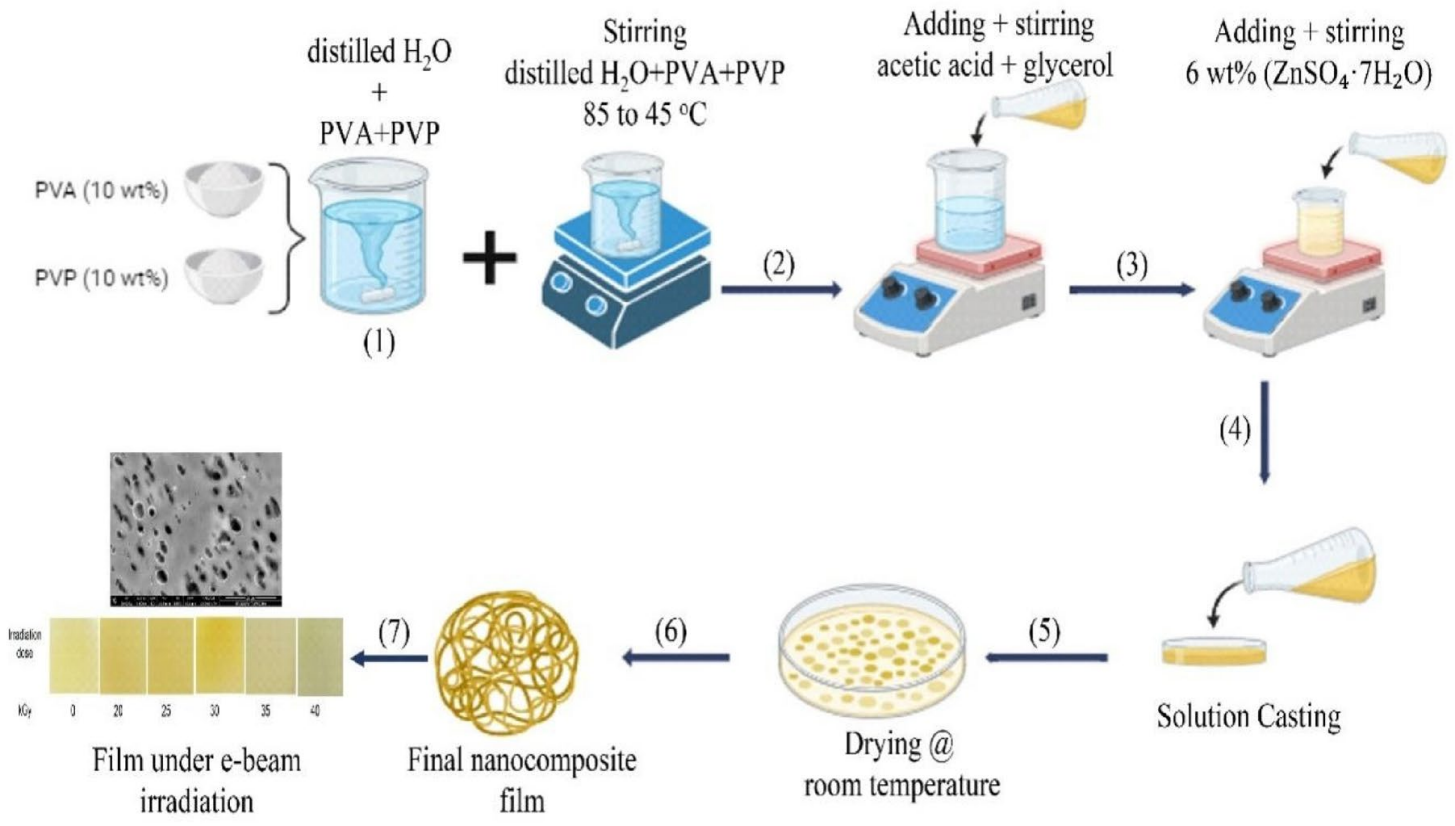
Preparation of PVA/PVP: ZnSO_4_.7H_2_O gel electrolyte polymer nanocomposite films.

Polymeric films with enhanced thermal and electrical conductivity were fabricated using a PVA/PVP blend. To prepare the blend, 10 wt% of PVA was dissolved in distilled water at 85 °C, while 10 wt% of PVP was separately dissolved at 45 °C. Subsequently, the two solutions were combined with a ratio of 40% PVA and 60% PVP. During stirring, acetic acid 14% (v/v) and glycerol 10% (v/v) were added to the blend to introduce carboxylic groups (COOH) for functionalization. Subsequently, 6 wt% of Zinc sulfate (ZnSO_4_·7H_2_O) was incorporated to the mixture as shown in scheme 1. Once fully dissolved, the resulting mixture was then cast into Petri dishes for film formation (solution casting) and left to dry at room temperature. The dried samples were irradiated using an electron beam at different doses in the range of 20–40 kGy. The electron beam was chosen for irradiation due to its more controlled impact on the physical properties, such as mechanical and thermal properties, compared to gamma rays. The thickness of the resulting films was measured at approximately 1.8 mm using a digital micrometer screw gauge. Furthermore, the structural formation process of PVA/PVP doped with Zn^2+^ nanostructures proceed as follows:

When ionizing radiation interacts with aqueous media, it induces water radiolysis, leading to the formation of several reactive species. A portion of the absorbed energy is converted into highly reactive intermediates according to:

$${H_2}O{\text{ }} \to {\text{ }}{e_{aq}},{\text{ }}{H^ \bullet }{,^ \bullet }OH,{\text{ }}{H_2},{\text{ }}{H_2}{O_2},{\text{ }}{H_3}{O^+},{\text{ }}O{H^ - },{\text{ }}H{O_2}$$

This process generates short-lived radicals (H^•^, ^•^OH) and solvated electrons (e_aq_), which act as powerful reducing and oxidizing agents, respectively. In irradiated aqueous solutions containing zinc sulfate precursor (ZnSO_4_), these reactive species initiate the reduction of Zn^2+^ ions, ultimately leading to the formation of zinc nanoparticles.

In the present synthesis, glycerol was incorporated to enhance the reducing environment. Upon reaction with ^•^OH or H^•^ radicals, glycerol forms hydroxyalkyl radicals, which also function as strong reducing agents:

$${R_2}CHOH\,+\,H{(^ \bullet }OH) \to {R_2}^{ \bullet }COH$$

Additionally, acetic acid was introduced as a scavenger for ^•^OH and H^•^ radicals to improve the reduction efficiency of the system:

$$HCO{O^ - }{+^ \bullet }OH/{H^ \bullet } \to CO{O^ - }+{\text{ }}{H_2}O/{H_2}$$

Hydroxyalkyl radicals may further react with ^•^OH or H^•^ via hydrogen abstraction, generating secondary radicals and molecular products:

$${R_2}^{ \bullet }COH{\text{ }}{+^ \bullet }OH/{H^ \bullet } \to {\left( {C{H_3}} \right)_2}COH{\text{ }}+{\text{ }}C{H_2}CHOHC{H_3}\,+\,{H_2}O/{H_2}$$

The reactive intermediates produced in these steps—including e_aq_, COO^–^, and (CH_3_)_2_COH radicals-contribute to the stepwise reduction of zinc ions:

$$Z{n^{2+}}+{\text{ }}{e^ - } \to Z{n^+} \to Z{n^0}$$

$$Z{n^+}+{\text{ }}CO{O^ - } \to {\left[ {Z{n^+}COO} \right]^{n - 1}} \to Z{n^{n - 1}}+{\text{ }}C{O_2}$$

$$Z{n^+}+{\text{ }}{\left( {C{H_3}} \right)_2}COH \to {\left[ {Zn{{\left( {C{H_3}} \right)}_2}COH} \right]^{n+}} \to Z{n^{n - 1}}+{\text{ }}{\left( {C{H_3}} \right)_2}C\,=\,O\,+\,{H^+}$$

Subsequent reactions between intermediate zinc species promote further reduction and nucleation:

$$2Z{n^{\left( {n - 1} \right)+}} \to {\text{ }}Z{n^+}+{\text{ }}Z{n^{\left( {n - 2} \right)+}}$$

$$2Z{n^+}+{\text{ }}2O{H^ - } \to {\text{ }}2\left[ {ZnOH} \right]{\text{ }} \to {\text{ }}2ZnO\,+\,{H_2}O$$

The presence of stabilizing polymers such as polyvinyl alcohol (PVA) and polyvinylpyrrolidone (PVP) plays a crucial role in controlling nanoparticle formation. These polymers prevent particle aggregation caused by high surface energy through interactions between their functional groups (–OH, –NH_2_, –CONH_2_, –COOH) and metal atoms or ions. The lone pair electrons of these functional groups coordinate with the nanoparticle surface, forming stabilizing covalent or coordination bonds.

Moreover, steric hindrance from the polymer chains provides an additional physical barrier that suppresses agglomeration. Through combined electrostatic and steric stabilization mechanisms, repulsive forces counterbalance attractive interactions, ensuring colloidal stility. These polymers also act as capping agents, regulating nanoparticle size and protecting against oxidation or aggregation during and after synthesis. For effective control of reduction and stabilization, such agents are introduced prior to irradiation.

### Sample irradiation

Each sample was irradiated with a controlled electron beam dose in the range of 20–40 kGy under ambient conditions at 25 °C. The irradiation was performed using an industrial linear accelerator developed at the National Center for Radiation Research and Technology (NCRRT), Egyptian Atomic Energy Authority, Cairo, Egypt. The accelerator operated at electron energy of 3 MeV with a peak beam current of 3 mA. Precise regulation of the absorbed dose and dose rate, fixed at 3 kGy min^-1^, was maintained throughout the irradiation process, with a conveyor speed of 16 m min^-1^. The absorbed radiation dose was measured using a primary standard graphite calorimeter.

### Measurements

The profile of XRD (XRD) of the polymer incorporate films were acquired at room temperature with a Philips EXPERT-MPDUG PW-3040 diffractometer, Measurements were carried out over a 2θ range of 5° to 80° with CuK_α_ radiation (λ = 0.15406 nm), operating at 40 kV and 20 mA. The data were collected with a scanning speed of 20°/min and an incremental step size of 0.05°. The structural phases of samples were analyzed using Fourier transform infrared (FTIR) spectrum recorded at room temperature, and over wavenumber range of 4000 to 400 cm^-1^ at a scanning speed of 16 cm/s using a Bruker-FTIR-ALPHA II spectrophotometer equipped with an ATR unit. Thermal behavior was assessed via differential scanning calorimetry (DSC) and thermogravimetric analysis (TGA) using a Labsys Evo TGA/DTA/DSC instrument (Setaram) under a nitrogen atmosphere, with α-alumina serving as the reference. The samples were heated from ambient temperature up to 700 °C at a constant rate of 10 °C/min. The elemental composition of the prepared films was examined using energy-dispersive X-ray spectroscopy (EDXS) to identify and quantify the constituent elements present in the samples. Surface morphology was characterized using a field emission scanning electron microscope (FE-SEM), model FEG Quanta 250. Prior to imaging, a thin gold coating was applied to the samples via sputter coating to reduce charging artifacts caused by the interaction with the electron beam.

Impedance spectroscopy and dielectric measurements were performed across a frequency range of 0.1 kHz to 1.0 MHz, and a temperature range of 303–373 K, using an oscillating voltage amplitude of 10 mV. These measurements were automated using a FLUKE PM6306 RLC bridge. Sample conductivity was assessed by sandwiching the sample between two stainless-steel electrodes (surface area of 0.1 m²) connected to the LCR meter system.

## Results and discussion

### Structure characteristics

#### X-ray diffraction (XRD) analysis

**Fig. 1:**
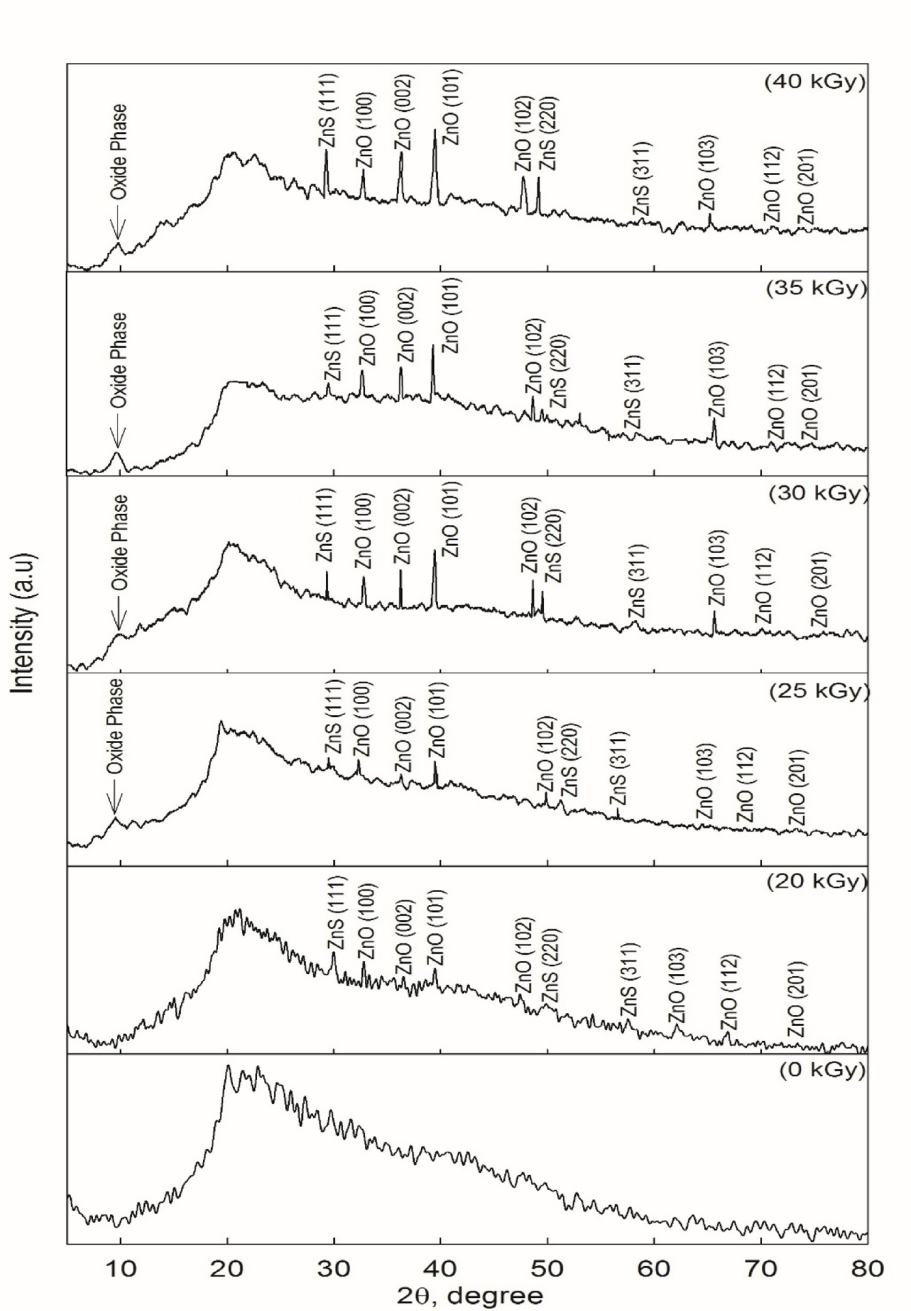
XRD spectra recorded for the investigated nanocomposite films and irradiated at doses of 0, 20, 25, 30, 35, and 40 kGy.

Figure 1 shows the measured x-ray diffraction pattern for investigated PVA/PVP films doped with Zinc Sulfate (ZnS) nanoparticle. In this pattern and for non-irradiated sample, the XRD spectrum shows a hump centered in the range 2θ = 15^o^−45^o^ accompanied by no detectable peaks, indicating non-crystalline characteristics of non-irradiated films. An increase in the irradiation dose by 20 kGy results in the emergence of diffraction peaks at 19.48^o^ to 23.33^o^ which corresponds to poly-crystalline interaction between hydroxyl (-OH) and carbonyl (C = O) functional groups of PVA and PVP^33^.

A subtle shift in the amorphous peak position near ~ 10° is attributed to the presence of PVP molecules^33^. Additionally, distinct peaks at 2θ = 29.62°, 49.40°, and 57.67° are observed, corresponding to the (111), (220), and (311) planes of ZnS nano-crystallites with a wurtzite structure (JCPDS: 36–1450)^33^. Other notable peaks at 2θ = 31.92°, 36.16°, 39.0°, 48.88°, 64.64°, 69.2°, and 73.42° represent the (100), (002), (101), (102), (103), (112), and (201) planes of ZnO, which also exhibit a hexagonal wurtzite structure (JCPDS: 36–1451)^33^. In Wurtzite ZnO has a hexagonal structure, in which each zinc atom is tetrahedral, coordinated with four oxygen atoms, and each oxygen atom is similarly bound to four zinc atoms, forming tetrahedral ZnO units. This arrangement results in two distinct polar faces along the c-axis of the hexagonal structure. One face consists solely of Zn^2+^ ions, while the opposite face is composed entirely of O^2-^ ions. These polar faces create an inherent asymmetry in the crystal, which contributes to the piezoelectric and pyroelectric properties of ZnO, making it valuable for various electronic and sensor applications^34^. With increasing e-beam doses above 20 kGy, the intensity of these diffraction peaks increases, indicating a tendency of the studied films to form greater crystallinity. The e-beam energy facilitates the alignment of crystallites into a more stable configuration, promoting crystallinity. The sharp diffraction peaks are associated with the hexagonal wurtzite structures of ZnO and ZnS, reflecting a well-ordered distribution of crystallites and complex particle formation within the material. This structural complexity is argued to the interaction between ZnO/ZnS nanoparticles and the amorphous regions of the PVA/PVP blend, contributing to the growth of the crystalline phase^34^. Moreover, the interaction between the PVA/PVP backbone and ZnO/ZnS nanoparticles involves the carbonyl (C = O) and hydroxyl (-OH) groups, with PVP playing a role in stabilizing the system by reducing intermolecular interactions through coordination with the carbonyl groups^34^.

#### FT-IR spectroscopy

**Fig. 2:**
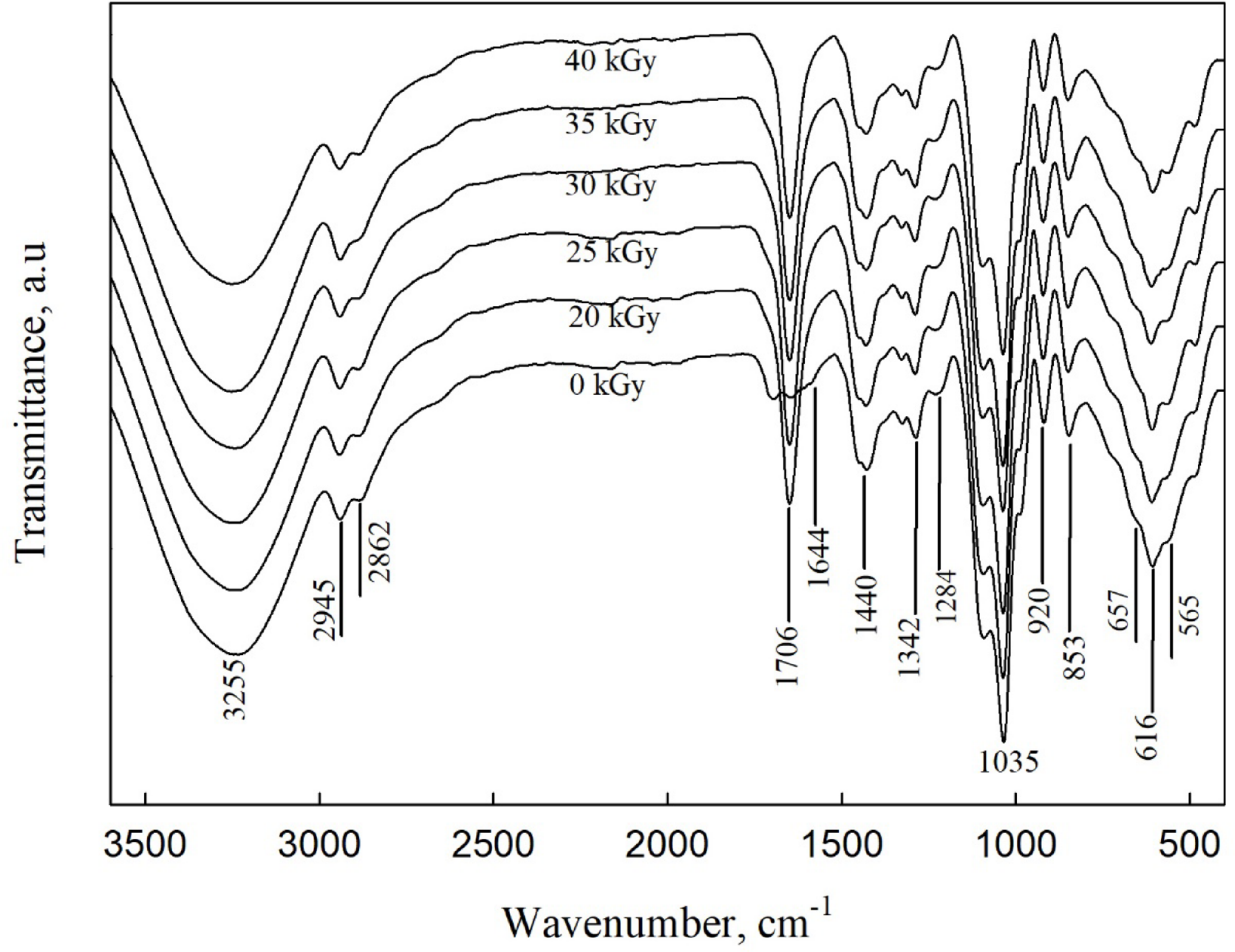
The investigated FTIR spectra are conducted to determine the effects of electron beam irradiation on PVA/PVP/ZnSO₄·7 H₂O nanocomposite samples. The samples were irradiated within a dose range of 0 to 40 kGy.

Fourier transform infrared (FT-IR) analysis is an analytical method used to identify the functional structures within fabricated polymeric films. This analysis focuses on the influence of different additives and functional groups. This method provides valuable insights into crosslinking, interactions between components, and complexation within the composites. The functional groups in both non-irradiated and irradiated PVA/PVP films doped with zinc sulfate heptahydrate (ZnSO_4_·7H_2_O) nanoparticles (NPs) were defined by assessing the FTIR spectrum, as depicted in Fig. 2. The vibrational mode at 565 cm^-1^, associated with Zn–O bonding, is evident across all functional groups in the films^35^. The observed peaks at 616 and 657 cm^-1^ are attributed to Zn–S bonds, confirming the formation of ZnS particles^36^. The appearance of these peaks indicates the introduction of Zn^2^⁺ nanoparticles within the PVA/PVP matrix. Moreover, the figure displays a broad absorption band centered around 3255 cm^-1^ in the pure PVA/PVP film, which corresponds to the O–H stretching vibrations associated with hydroxyl groups present in the polymer blend^37^. The absorption bands at 2862 cm^-1^ and 2945 cm^-1^ correspond to C–H asymmetric and symmetric stretching vibrations, respectively^36–38^. The vibrational band at 1706 cm^-1^ is attributed to C = O stretching, while O–H bending vibration is detected at 1644 cm^-1^^38^ confirming intermolecular interactions between OH groups in PVA and carbonyl groups in PVP^39^. It was observed that the intensity of this band (1644 cm^-1^) increases against irradiation doses. Peaks at 1440 and 1342 cm^-1^ correspond to symmetric bending of CH_2_ in PVA and C–H wagging in PVP, respectively^39^. The C-N symmetric stretching results in an absorption peak at 1286 cm^-1^, while the C–O stretching of PVA appears at 1035 cm^-1^^40^. Additionally, bands at 920 and 853 cm^-1^are associated with C–C stretching vibrations^38,39^. The formation of hydrogen bonding observed between OH groups in PVA and carbonyl groups in PVP of the investigated sample are also detected between PVA/PVP blends doped with sericin synthetic polymer^41^.

**Fig. 3:**
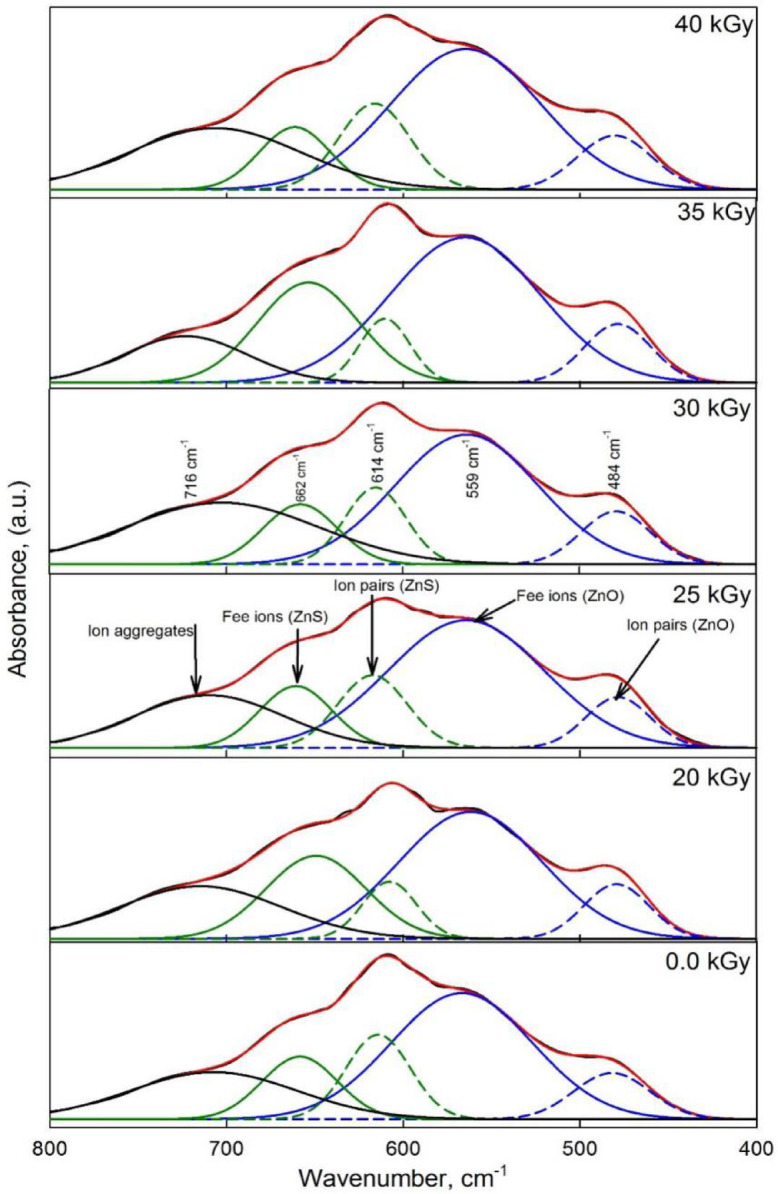
Deconvolution of the band between 400 cm^-1^ and 800 cm^-1^ of e-beam irradiated gel electrolyte polymer. The area under the blue dashed line (– –) corresponds to ion pairs of ZnO, while the solid blue line (―) represents free ions of ZnO. The green dashed line (– –) indicates ion pairs of ZnS, and the solid green line represents free ions of ZnS. The solid bulk line corresponds to aggregates. The red solid line represents the fitted band matching the experimental spectrum.

FTIR analysis was conducted to explore the transport parameters of charge carriers in the nanocomposite films. Figure 3 presents the FTIR spectral deconvolution performed at room temperature within the wavenumber range of 400 to 800 cm^-1^. The peaks observed at 484 cm^-1^ and 614 cm^-1^ are attributed to ion pair vibrations associated with ZnO and ZnS, respectively. The wavenumber at 559 cm^-1^ correspond to free ions of ZnO and at 662 cm^-1^ is free ions of ZnS, while 716 cm^-1^ correspond to ion aggregates. The area under the deconvolution peak for ion pairs (*A*_*p*_), free ions (*A*_*f*_) and ion aggregates (*A*_*a*_) of the nanocomposite films were determined using the following equations^40^.

1 $$\begin{gathered} Percentage~of~ion~pairs\left( \% \right)=\frac{{{A_p}}}{{{A_f}+{A_p}+{A_a}}} \hfill \\ Percentage~of~free~ions~\left( \% \right)=\frac{{{A_f}}}{{{A_f}+{A_p}+{A_a}}} \hfill \\ Percentage~of~ion~aggregates~\left( \% \right)=\frac{{{A_a}}}{{{A_f}+{A_p}+{A_a}}} \hfill \\ \end{gathered}$$

The calculated data using Eq. (1) are tabulated in Table 1 as function of applied irradiation doses. Indeed, the results indicate a decrease in ion pairs accompanied by an increase in free ions with increasing irradiation. As irradiation continues to increase both ion pairs and aggregates gradually decline in the nano-composite system. At the molecular level, several changes occur when the nanocomposite system is subjected to higher doses of irradiation. Irradiation energy disrupts these interactions by breaking the bonds that maintain ion pairs and aggregates resulting in more free ions as the dose increases^42^. Additionally, irradiation generates free radicals in the polymer matrix, which can trigger chemical reactions that promote crosslinking. This crosslinking reduces the mobility of ions, limiting their ability to form new pairs or aggregates.

**Table 1:** Percentage of ion pairs, free ions of ZnO and ZnS and percentage ion aggregates calculated using Eq. (1) as function of irradiation dose of the studied polymer films.

Irradiation doses, kGy	ion pairs %		Free ions %		ion aggregates %
	ZnO	ZnS	ZnO	ZnS	
0.0	8.93	13.79	42.98	9.55	27.41
20	8.78	13.16	43.68	10.25	22.80
25	8.69	12.39	45.31	11.05	20.45
30	8.56	10.81	45.78	12.01	19.94
35	8.52	7.41	48.77	19.87	19.37
40	8.04	7.11	49.12	22.57	12.75

#### Field emission scanning electron microscope (FE-SEM)

**Fig. 4:**
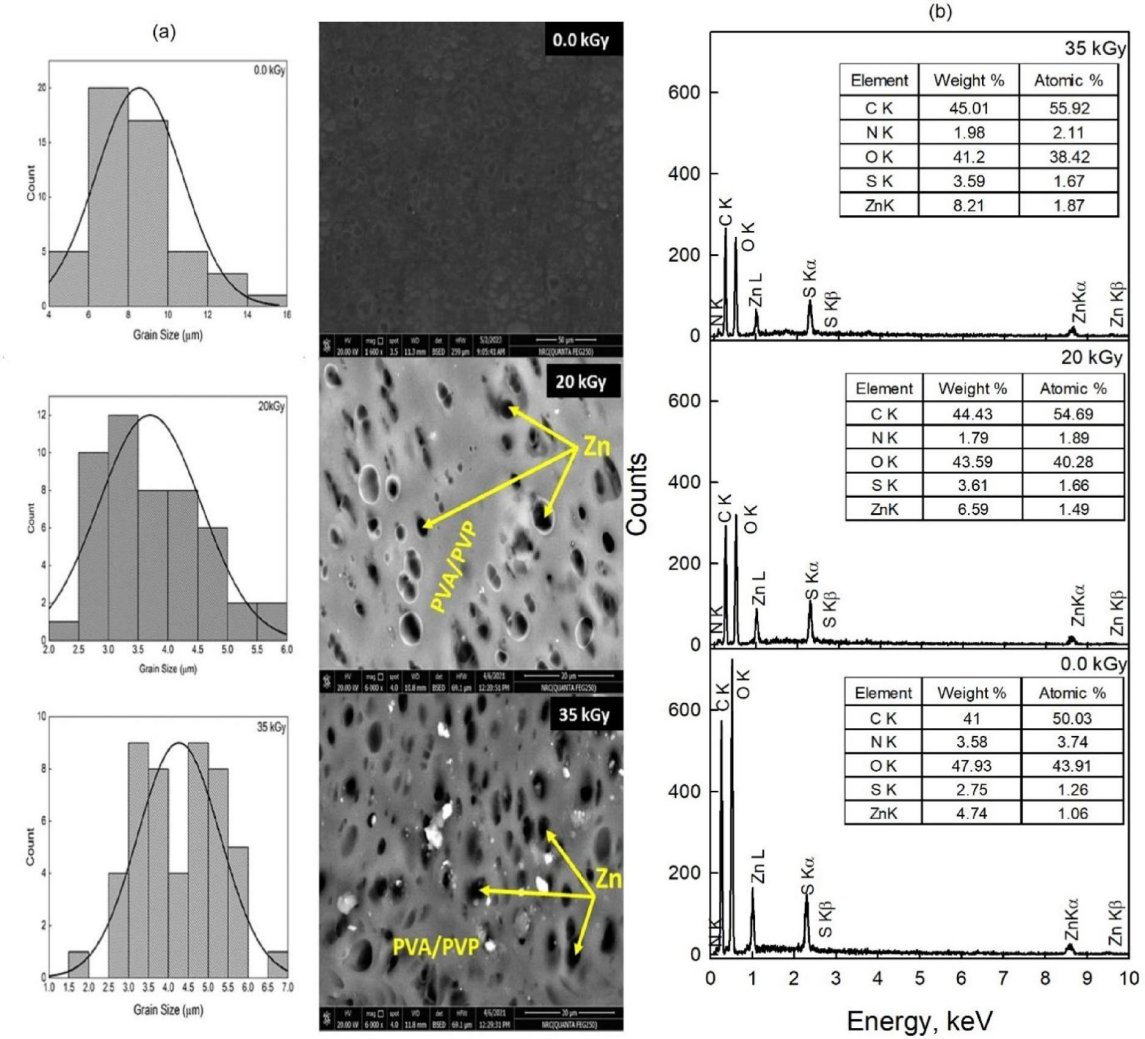
(**a**) Field emission scanning electron microscopy (FESEM) images, (**b**) The EDXS pattern of the nanocomposite films irradiated by e-beam at doses 0, 20, and 35 kGy.

FE-SEM micrographs of PVA/PVP containing zinc nano-particles at the irradiation dose 0.0, 20, and 35 kGy are presented in Fig. 4a. Regarding the non-irradiated samples (dose = 0.0 kGy) its FE-SEM image shows a smooth surface besides regularly and uniformly dispersed pores within the PVA/PVP network. On the other hand, increasing the irradiation dose to 20 kGy resulted in a much denser network structure, compacting molecular chains together and making more entanglements with fewer pores on the surface. At 35 kGy, FE-SEM presents a uniform distribution of semi-sphere zinc nanoparticles inside the porous network of PVA/PVP/acetic acid/glycerin^37^. The histograms and FESEM micrographs presented in Fig. 4a illustrate the grain size distribution of the nanocomposite films. The average grain size for the representative composition was estimated using the linear intercept method via ImageJ software. In this approach, a straight line is randomly drawn across the FESEM image, and the total line length is divided by the number of intersected grains. An average grain size is then calculated based on approximately 50 individual measurements taken from various grains in the microstructure. With an increase in the irradiation e- beam doses the grain size increased from 3.58 (20 Gky) to 4.25 (35 kGy) µm. However, the average grain size measured from FESEM provides the grain size of the particles, which is composed of several united crystallites^43^. When nanocomposite films are exposed to electron beam irradiation, various processes can take place depending on the properties of the films and the irradiation conditions particularly affecting grain size. The following mechanisms explain the increase in grain size during e-beam exposure: (i) e-beam irradiation generates localized heating in the nanocomposite films, which enhances atomic diffusion. As the dose increases, more energy is deposited, raising the local temperature. This temperature rise boosts atomic mobility allowing smaller grains to merge into larger ones^44^. (ii) At lower doses (around 20 kGy) irradiation may create defects like vacancies or dislocations within the nanocomposite microstructure. However, at higher doses (e.g., 35 kGy), the additional energy helps to anneal these defects, relieving internal stresses and fostering grain coalescence as the microstructure stabilizes. (iii) At higher irradiation doses the absorbed energy can initiate recrystallization processes, where new grains form and grow as the material attempts to lower its free energy. Higher doses supply enough energy for grain boundary movement, further promoting grain growth through recrystallization^45^.

Energy-dispersive X-ray spectroscopy (EDXS) was employed to determine the elemental composition and structural constituents of the investigated samples. The EDXS spectra presented in Fig. 4b correspond to 0.4PVA/0.6PVP zinc sulfate nanocomposite films exposed to electron-beam irradiation at doses of 0.0 kGy, 20 kGy, and 35 kGy. The spectra exhibit a characteristic oxygen peak at approximately 0.5 keV, Zn peaks around 1 keV, and additional Zn signals in the 8.5–9.5 keV range. These features confirm the formation of ZnO nanoparticles, indicating the coexistence of two phases: ZnO and ZnS. Furthermore, a sulfur peak observed near 2.25 keV provides clear evidence for the formation of zinc sulfide (ZnS) nanoparticles^15^. The inset tables in Fig. 4b summarize the elemental weight% and atomic% values of Zn, O, and S for different irradiation doses. At 0.0 kGy, the values are: Zn [4.74, 1.06], O [47.93, 43.91], and S [2.75, 1.26]. Upon irradiation at 20 kGy, the corresponding values become Zn [6.59, 1.49], O [43.59, 40.28], and S [3.61, 1.66]. At 35 kGy, they change to Zn [8.21, 1.87], O [41.20, 38.42], and S [3.95, 1.67]. As illustrated in Fig. 4b, increasing the irradiation dose from 0 to 20 kGy enhances the sulfur peak intensity by approximately 31%, while the oxygen peak decreases by about 9%. This trend is accompanied by a noticeable increase in both the weight% and atomic% of Zn. When the irradiation dose is further increased to 35 kGy, the Zn content rises significantly (by nearly 39% compared to the non-irradiated sample), with only a slight reduction in sulfur intensity. These results indicate that electron-beam irradiation promotes the formation and growth of Zn-based nanoparticles, while progressively modifying the relative contributions of ZnO and ZnS phases within the nanocomposite matrix.

### Thermal analysis

#### Differential scanning calorimetry (DSC)

**Fig. 5:**
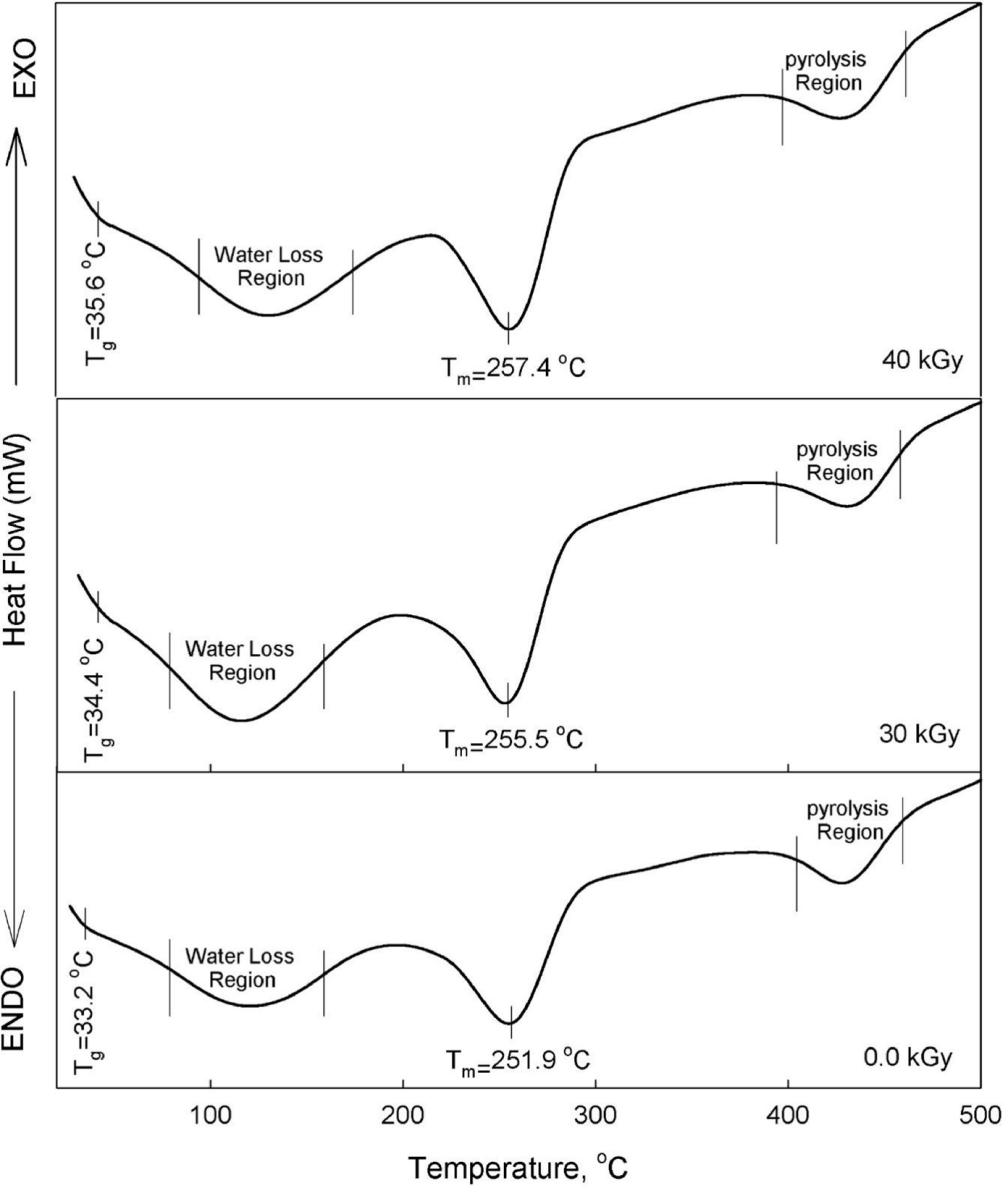
DSC thermos-grams of nano-composite films irradiated at doses 0, 30 and 40 kGy as a representative case. Samples at other doses has the same trend.

Thermal techniques are an effective method for analyzing physical and chemical changes, including phase transitions, glass transition temperature (*T*_*g*_), melting point (*T*_*m*_), and heat consumption (Δ*H*_*p*_). In the present study the investigated PVA/PVP samples doped with Zn^2+^ ions are thermally treated in the temperature range 30–700 °C with heating rate 10 °C/min under Nitrogen atmosphere using differential scanning calorimetry technique (DSC). The measured thermal spectrum as function of irradiation dose is shown in Fig. 5 at dose 25 kGy as a representative case. The endo thermic transitions *T*_*g*_ observed in the figure can be described as follows: For non-irradiated films and as listed in Table 2, *T*_*g*_ has a value of ~ 33.2 °C which increases to 35.6 °C against irradiation dose. This shift in *T*_*g*_ toward higher temperature indicates that the PVA/PVP molecular chains have become less mobile and more rigid. This change can be attributed to factors such as increased cross-linking, higher crystallinity, stronger intermolecular interactions (such as hydrogen bonding), or the addition of certain fillers or additives. Consequently, the polymer requires more thermal energy to allow its molecular chains to transit from a rigid, glassy state to a more flexible, rubbery state^46^. The broad peak in the temperature ranges from **~** 75 to 150 °C is due to the release of water which may be assigned to the relaxation associated with the crystalline regions^46^.

**Table 2:** Differential scanning calorimetry (DSC) parameters of irradiated PVA/PVP/Zn^2+^ gel polymer electrolyte, including glass transition temperature (T_g_), melting temperature (T_m_), melting enthalpy (ΔH_m_), degree of crystallinity (Q_c_), and heat consumption (ΔH_p_).

Irradiation dose, kGy	Glass phase transition T_g_ ◦C	Melting phase transition		Crystallinity Q_c_ %	Thermal pyrolysis ΔH_*p*_ J/g
		T_m_ ◦C	ΔH_m_ J/g		
0.0	33.2	251.9	8.6	6.2	121.5
20	33.8	253.2	21.8	15.7	241.1
25	33.9	254.3	19.2	13.8	136.2
30	34.3	255.5	26.32	19.0	177.2
35	34.6	255.5	37.82	27.3	139.9
40	35.6	257.4	38.2	27.5	186.6

The endothermic melting temperatures (*T*_m_) values of the nanocomposite films, which increase with higher irradiation doses, are added to Table 2, and indicate enhanced thermal stability. The *T*_m_ values, which vary with doses, suggest that even a small amount of Zn^2+^ dispersed in the PVA/PVP blend matrix significantly boosts the nanocomposite films thermal stability. Typically, the melting temperature helps to identify a substance’s nature and its purity. However, in these films, changes in the shape and area of *T*_m_ endothermic peak also reflect the degree of crystallinity and the strength of polymer-nano-particle interactions. These interactions can alter the crystalline structure, which in turn affects the melting enthalpy (Δ*H*_m_) during the phase change. The Δ*H*_*m*_ values for the nano-composite films, obtained from the integrated area under the DSC endothermic melt transition peak, are added Table 2. The crystallinity (*Q*_c_) of the films is calculated using the formula *Q*_c_ = Δ*H*_*m*_/$\:\varDelta\:{H}_{m}^{0}$^9,48^, where $\:\varDelta\:{H}_{m\:\:}^{0}$is 138.6 J/g for a 100% crystalline PVA structure^46^. The evaluated *Q*_c_ values which are inserted in Table 2 shows an increase versus e-beam irradiation doses. The unusual variation in *Q*_c_ versus irradiation doses (in crystalline materials) in the PVA-PVP blend confirms significant changes in polymer-polymer and polymer-nanoparticle interactions due to irradiation which consistent with the XRD, and FESEM, results for the present polymer nanocomposites. The rightmost large endothermic peak, observed in the temperature range of approximately 400 to 450 °C, corresponds to the thermal pyrolysis of the films. Thermal pyrolysis is the process where the material undergoes thermal decomposition in the absence of oxygen, breaking down into smaller molecules. The endothermic nature of this peak indicates that the process absorbs heat from the surroundings, which is typical during the breakdown of chemical bonds in the structure. The amount of heat absorbed during this process is represented by the heat consumption, denoted as (*ΔH*_*p*_) which quantifies the energy required for the pyrolysis to occur, reflecting the stability and decomposition characteristics of the film material at high temperatures^46^.

#### Thermogravimetric analysis (TGA)

Thermal analysis helps also to understand the relaxation processes that occur in polymeric materials. There are two types of relaxation: α-relaxation and β-relaxation. α-relaxation also known as the glass transition (*T*_g_), is related to the side (functional) groups of polymers. On the other hand, *β*-relaxation is associated with the segmental motion of the polymer main chain. The derivative thermogravimetric (DTG) analysis is a valuable tool for identifying the various stages of thermal degradation of chemical species, including macromolecules, in the doped polymeric sample.

**Fig. 6:**
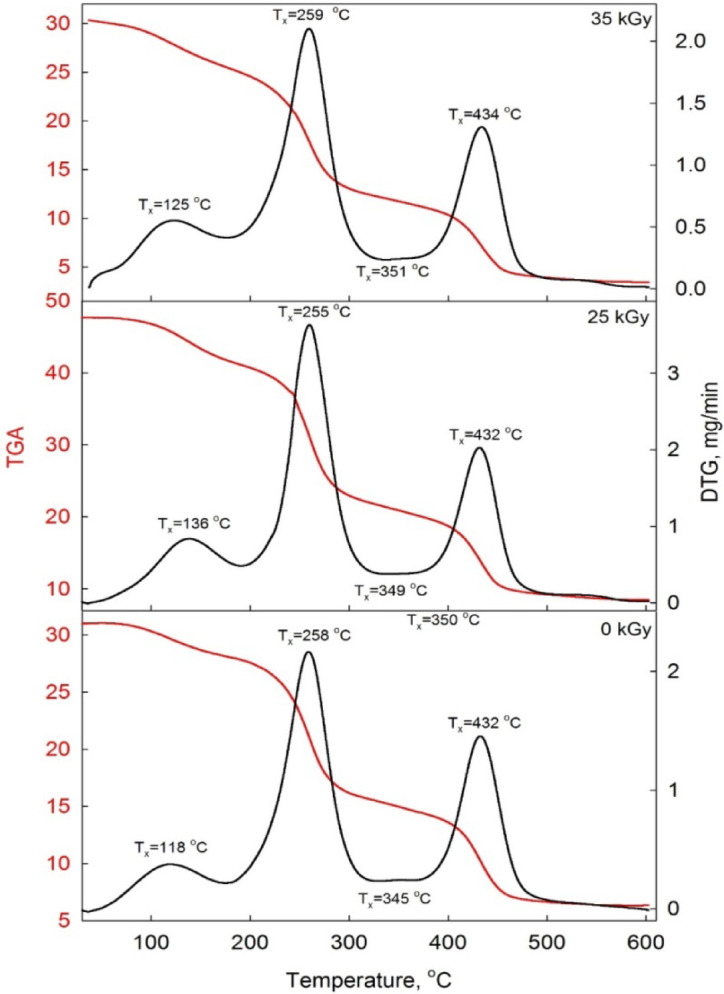
The thermogravimetric analysis (TGA) spectrum (red line) and its derivative thermogravimetric curve (DTG) (black line) for the studied PVA/PVP/Zn^2+^ at irradiation doses of 0, 25, and 35 kGy.

Thermogravimetric Analysis (TGA) is a crucial technique for understanding the thermal properties and stability of the material. Figure 6 shows the TGA thermos-grams for the investigated PVA/PVP/Zn^2+^ nanocomposite at doses 0.0, 25 and 35 kGy. The thermal degradation shows four main stages. The initial weight loss observed at lower temperatures (**~** below 150 °C) is usually attributed to the evaporation of absorbed moisture and any residual solvents in the material. PVA and PVP are both hygroscopic, which can result in significant weight loss during this phase^47^.

The second stage is Polyvinyl alcohol (PVA) generally begins to decompose around 150–290 °C, with significant weight loss corresponding to the breakdown of the polymer hydroxyl groups and main chain^49^. The third stage is Polyvinyl pyrrolidone (PVP) typically decomposes at a higher temperature, around 300–460 °C^48^. The final residue at high temperatures represents an inorganic content, primarily from the Zn^2+^ ions or any remaining zinc compounds that do not decompose. Higher residue percentage indicates the presence of a significant amount of inorganic material, which could contribute to the nanocomposite overall thermal stability. In addition, if the nanocomposite has been exposed to electron beam irradiation, thermogravimetric analysis can be utilized to discern the effects of this treatment on the material thermal properties. Irradiation can induce crosslinking or cause changes in the polymer matrix potentially leading to shifts in the decomposition temperature and changes in the overall thermal stability. The TGA curve will show a second major weight loss step corresponding to the degradation of PVP. The presence of Zn^2+^ ions within the PVA/PVP matrix can alter the thermal stability of the nanocomposite. These ions might interact with the polymer chains, potentially enhancing the thermal stability by increasing the decomposition temperature or modifying the decomposition pathway^49^.

#### Evaluation of thermogravimetric kinetic parameters

Several methods are utilized to calculate and analyze the kinetic parameters of the pyrolysis process, including the differential method proposed by Freeman and Carroll^50^, the integral method developed by Coats and Redfern^51^, and the approximation method introduced by Horowitz and Metzger^52^. The obtained results from these methodologies demonstrate the absence of substantial variation in the kinetic parameters. This observation indicates that these techniques can be utilized interchangeably. The general form of the rate equation for non-isothermal kinetic studies, frequently involves the decomposition of materials under a controlled increase in temperature. The rate of degradation or conversion, $\:\frac{d\phi\:}{dt}$​, can be written in terms of a temperature-dependent rate constant *k*(*T*) and the function $\:f\left(\phi\:\right)$, which represents the dependence of the rate on the degree of conversion . The equation is typically written as^9,48^:

2 $$\frac{\mathrm{d}\phi}{\mathrm{d}t}=kf(ϕ) $$

Where $f\left( \phi \right)$ is reaction model function of conversion and can be represented as $f\left( \phi \right) = (1 - \phi )^{r}$, where *r* is the reaction order, $\phi$ denotes the reacted fraction of the thermally treated material and is given by$\phi = {\text{ }}\left( {m_{i} {-}m_{t} } \right)/{\text{ }}\left( {m_{i} {-}m_{f} } \right)$, *m*_*t*_ is the mass at an instant of time *t* (during the decomposition process), *m*_i_ denote the is initial mass of sample and *m*_*f*_ is final (residual) mass, at the end of a particular decomposition stage of the material (under influence of heat). Generally, *k* could be written in Arrhenius equation form as *k* = *A* exp(-*E*_*th*_/*RT*), where *E*_*th*_ is the activation energy of thermal transformation, *A* represents the pre-exponential factor and *R* is the universal gas constant^48^. The rate of heating $\beta$ could be determined by dividing temperature, *T*, at which the reaction occurs at a given time, *t*, i.e., $\beta = dT/dt$. However, *d*$\:{\phi\:}$*/dt* in Eq. (2) could be written as $\:\frac{d{\phi\:}}{dT}\frac{dT}{dt}=\beta\:\frac{d{\phi\:}}{dT}$, where $\:\frac{d{\phi\:}}{dT}$ is given by:

3 $$\:\frac{d{\phi\:}}{dT}=\left(\frac{A}{\beta\:}\right)exp\left(\frac{-{E}_{th}}{RT}\right){(1-{\phi\:})}^{r}$$

To calculate the kinetic parameters - thermal activation energy *E*_th_​ and the pre-exponential factor A the following fundamental relationship is commonly used in thermal analysis^48^:

4 $$\:G\left({\phi\:}\right)={\int\:}_{0}^{T}\left(\frac{A}{\beta\:}\right)exp\left(-\frac{{E}_{th}}{RT}\right)dT\:\:\:\:\:\:$$

Therefore, the kinetic mechanism equation can be simplified as follows:

5 $$\:{ln}\left[G\left({\phi\:}\right)\right]=\left(-\frac{{E}_{th}}{RT}\right)+ln\left(\frac{AR}{\beta\:{E}_{th}}\right)\:\:$$

Where the function *G*($\:{\phi\:}$) is given by:

6 $$G\left( \phi \right)= - \frac{{\ln \left( {1 - \phi } \right)}}{{{T^2}}}~~\left( {for~r=1} \right),~\,or\,~G\left( \phi \right)= - \frac{{1 - {{\left( {1 - \phi } \right)}^{1 - r}}}}{{\left( {1 - r} \right){T^2}}}~~\left( {for~r \ne 1} \right)$$

Based on Eq. (5), a plot of *ln*[*G*($\:\phi\:$)] vs. 1/*T* yields a straight line from which the activation energy $\:{E}_{th}$ and pre-exponential factor *A* can be calculated from the slope and intercept, respectively. Enthalpy is a state function that represents the heat absorbed or released during a chemical reaction under constant pressure. It also reflects the energy required for further chemical bond dissociation. As the temperature increases, more thermal energy is supplied to the samples, resulting in the dissociation of relatively strong chemical bonds. The enthalpy of thermal degradation is calculated using the equation: Δ*H*_*tg*_ = *E*_*th*_- *RT*_*x*_, *T*_*x*_ *i*s the optimum temperature for degradation. Furthermore, *T*_*x*_ ​ is defined as the temperature corresponding to the maximum rate of mass loss, which can be identified from the peak position in the negative region of the differential thermogravimetric (DTG) as shown in Fig. 6. In other words, it is the temperature at *m/m*_*i*_ *=1/e*^31^. It should be noted that the first derivative DTG of measured decomposition curves are used to determine the temperature corresponding to maximum rate of degradation, *T*_*x*_. In addition, the Gibbs free energy, $\:\varDelta\:G$, can be obtained using activation energies and pre-exponential factors^9,48^ as $\:\varDelta\:G={E}_{th}+R{T}_{x}ln\left(\frac{{k}_{B}{T}_{x}}{hA}\right)$, where *k*_*B*_ is the Boltzmann constant, and *h* is the Plank constant. Furthermore, the entropy could be determined by using the following equation $\:\varDelta\:S=\frac{{\varDelta\:H}_{tg}-\varDelta\:G}{{T}_{x}}$^48^. The assessed values of *T*_*x*_ besides activation energy (*E*_*th*_), pre-exponential factor (*A*) besides enthalpy (*ΔH*_*tg*_), Gibbs free energy (*ΔG*) and entropy *ΔS*) are listed in Table 3 for the nanocomposite films at various irradiation doses are inserted in Table 3. Different reaction orders (*r* = 0.5, 1, 2, and 3) were considered to evaluate the kinetic parameters using Eqs. (5) and (6). Linear best-fit analysis was applied to identify the most appropriate reaction order for each case. The data in Table 3 indicate that *r* = 2 provide the best fit for the samples, with correlation coefficients (*R*²) close to 0.9999. Both ‘*E*_*th*_’ and ‘*A*’ values increase against irradiation doses at all stages of thermal decomposition, suggesting an improvement in the thermal stability of the gel electrolyte polymer nanocomposite films. Table 3 illustrates that the activation energy (*E*_*th*_) rises with increasing doses of electron beam irradiation. This irradiation notably modifies the molecular structure of polymers, thereby improving their thermal stability and raising the activation energy required for thermal degradation. This occurs primarily through two mechanisms: cross-linking and molecular organization. Cross-linking involves the formation of chemical bonds between individual polymer chains, strengthening the overall molecular network. This added structural integrity requires more energy to break these reinforced bonds, thereby increasing the activation energy for decomposition and other reactions^48^. Additionally, irradiation can lead to a more ordered molecular structure, aligning or organizing polymer chains in a way that reduces free volume and enhances intermolecular forces, such as van der Waals interactions. This organized structure restricts chain mobility, making it more challenging for thermal energy to initiate bond-breaking or molecular motions, further raising the activation energy. As a result, irradiated polymers display improved resistance to thermal degradation, requiring higher temperatures or longer heat exposure to decompose compared to their non-irradiated counterparts^49,50^. The pre-exponential factor, represented as (*log A*), is a parameter in the Arrhenius equation and plays an important role in determining the combustibility of a nanocomposite films. This factor reflects the frequency of effective molecular collisions, meaning it represents how often molecules collide with sufficient orientation and energy to initiate a reaction, such as combustion^48^. The change in enthalpy (Δ*H*_tg_) represents the energy difference between the reactants in their ground state and the activated reaction center, which corresponds to the activation energy needed to drive the reaction. This enthalpy difference indicates the amount of energy the system must absorb or release to transition from the reactants to the activated state. When this energy difference is small, the formation of the reaction center is more favorable, as the lower energy barrier facilitates the reaction process^53^. As listed in Table 3, the positive Δ*H*_*tg*_ indicates that an external energy source is necessary to raise the energy level of the reactants. Additionally, higher enthalpy values suggest that the system is less reactive, as more energy is required to initiate the reaction. This implies that the system has a higher energy barrier to overcome before reaching the reaction center, leading to reduced reactivity. The Gibbs free energy (Δ*G*) reflects an increase in the total chemical energy within the process, signifying the advancement of the reaction and the establishment of the reaction center. A positive (*ΔG*) indicates that energy input is required for the reaction to proceed, while a negative (*ΔG*) suggests a spontaneous progression toward the reaction center formation. The increase in (*ΔG*) suggests that the system is moving toward a higher-energy state, which enables the initiation of critical reactions and the creation of active sites for chemical transformations. This shift arises from functionalization, which induces variations in entropy (*ΔS*) and involves detailed heat flow analysis (Δ*H*_t*g*_). A larger (Δ*G*) value typically correlates with a reaction that is less favorable, as it indicates a higher energy barrier. However, in the studied films, the elevated (Δ*G*) values suggest a lower energy barrier and minimal heat absorption during combustion, highlighting an efficient transition with reduced energetic demands^54^. The data presented in Table 3 indicate that the calculated entropy values (*ΔS*) are negative for all samples, which signifies a decrease in system disorder. This reduction in entropy suggests that the system is becoming more ordered, reinforcing the idea that bond dissociation results in a more organized structure compared to the original reactants. As the polymer chains rearrange and form stronger interactions, the overall molecular configuration becomes more stable and less chaotic, contributing to the enhanced structural integrity of the material^54^.

**Table 3:** Thermal and kinetic parameters of irradiated PVA/PVP/Zn^2+^ gel polymer electrolyte, including the temperature at the highest degradation rate (*T*_x_), temperature width of the 1^st^, 2^nd^, 3^rd^, and 4^th^ regions during thermal dissociation, reaction order (*r*), activation energy (*E*_*th*_), pre-exponential factor (*A*), fitting correlation coefficient (R²), enthalpy (*ΔH*_*tg*_), Gibbs free energy (*ΔG*), and entropy (*ΔS*).

Irradiationdose, kGy	T_x_, ^o^C	Temp. regions,^o^C		*r*	E_th_, kJ/mol.	Log A, s^-1^	ΔH_tg_ kJ/mol.	ΔG kJ/mol.	ΔS J/mol.
0.0	118	1^st^	54–195	2	26.60 ± 0.01	3.37	23.35	97.95	−190.78
	258	2^nd^	220–285		35.14 ± 0.01	3.39	30.73	133.14	−192.85
	345	3^rd^	285–411		75.32 ± 0.02	3.45	70.11	191.26	−193.22
	432	4^th^	412–460		145.37 ± 0.01	4.50	139.51	262.22	−174.05
20	136	1^st^	93–215	2	49.31 ± 0.07	3.18	45.91	125.56	−194.74
	260	2^nd^	245–280		55.39 ± 0.04	3.23	50.21	155.67	−169.26
	350	3^rd^	300–400		74.67 ± 0.03	4.07	69.49	173.27	−166.56
	432	4^th^	415–450		300.08 ± 0.06	5.35	294.22	405.50	−157.83
25	136	1^st^	70–213	2	70.02 ± 0.07	4.10	66.82	135.01	−176.67
	255	2^nd^	230–275		170.19 ± 0.02	4.94	165.81	252.02	−163.28
	349	3^rd^	300–380		133.87 ± 0.07	4.55	128.71	235.69	−172.00
	432	4^th^	410–450		246.06 ± 0.03	5.14	240.20	354.28	−161.81
30	115	1^st^	80–210	2	59.54 ± 0.04	3.83	56.32	126.88	−181.85
	259	2^nd^	240–277		169.61 ± 0.07	4.93	165.19	252.16	−163.48
	350	3^rd^	290–400		106.48 ± 0.03	4.24	101.30	212.17	−177.95
	437	4^th^	406–454		233.86 ± 0.02	5.08	227.96	343.66	−162.94
35	120	1^st^	90–213	2	57.30 ± 0.06	3.73	54.03	126.35	−184.01
	258	2^nd^	238–271		195.45 ± 0.03	5.08	191.04	276.28	−160.53
	348	3^rd^	308–395		133.65 ± 0.07	4.53	128.49	235.61	−172.48
	434	4^th^	416–453		221.28 ± 0.07	5.02	215.41	331.42	−164.08
40	125	1^st^	105–217	2	67.02 ± 0.04	3.97	63.72	135.14	−179.46
	259	2^nd^	237–278		174.64 ± 0.06	4.96	170.22	256.88	−162.89
	351	3^rd^	304–395		156.33 ± 0.06	4.74	151.15	256.30	−168.51
	434	4^th^	405–460		204.59 ± 0.07	4.93	198.72	315.92	−165.77

### Impedance spectroscopy analysis

The complex impedance, Z^*^(ω) of a material is expressed as^55^:

7 $$\:{Z}^{*}\left(\omega\:\right)={Z}^{{\prime\:}}+i{Z}^{{\prime\:}{\prime\:}}$$

denotes the real part of the impedance, which represents the resistive element that dissipates energy in the form of heat. Z'' is the imaginary part of the impedance, which is associated with the reactive or capacitive behavior of the material. In the context of impedance spectroscopy, these components are instrumental in providing critical information regarding dielectric relaxation processes. This is achieved by indicating the frequency-dependent response of the material’s polarization mechanisms.

The construction of an equivalent circuit model from the provided data facilitates the simulation of material behavior under various frequencies. This process unveils intricate details regarding the conduction, relaxation, and interfacial effects that manifest in polymers, polymer blends, and composites.

The dielectric constant ε′, dielectric loss ε′′, real electrical modulus M′, and imaginary electrical modulus M′′ can be derived from the complex impedance data Z^*^(ω) using the following relationships^55^:

8 $$\begin{gathered} {\varepsilon ^*}=\frac{1}{{i\omega {C_o}{Z^*}}}=\varepsilon ^{\prime} - ~i\varepsilon ^{\prime\prime} \hfill \\ {\varepsilon ^*}=\frac{1}{{i\omega {C_o}\left( {Z^{\prime} - ~iZ^{\prime\prime}} \right)}} \hfill \\ \end{gathered}$$

9 $$\:{\epsilon\:}^{{\prime\:}}=\frac{1}{\omega\:{C}_{o}}\left(\frac{{Z}^{{\prime\:}{\prime\:}}}{{\left({Z}^{{\prime\:}}\right)}^{2}+{{(Z}^{{\prime\:}{\prime\:}})}^{2}}\right)\:\:\:\:{\epsilon\:}^{{\prime\:}{\prime\:}}=\frac{1}{\omega\:{C}_{o}}\left(\frac{{Z}^{{\prime\:}}}{{\left({Z}^{{\prime\:}}\right)}^{2}+{{(Z}^{{\prime\:}{\prime\:}})}^{2}}\right)$$

In this equation, C_o_ is equivalent to ε₀A/d, where ε₀ signifies the permittivity of free space, A denotes the area of the structure in which the electrolyte is mounted between electrodes, and d represents the thickness of the sample. The component, denoted by the symbol ω, is equivalent to 2πf, where f represents the frequency. The electrical modulus is the reciprocal of the complex permittivity^55^:

10 $$M^{\prime}=\left( {\frac{{\varepsilon ^{\prime\prime}}}{{{{\left( {\varepsilon ^{\prime}} \right)}^2}+{{({\varepsilon ^{''}})}^2}}}} \right)\,\,\,\,\,\,\,M^{\prime\prime}=\left( {\frac{{\varepsilon ^{\prime}}}{{{{\left( {\varepsilon ^{\prime}} \right)}^2}+{{({\varepsilon ^{''}})}^2}}}} \right)$$

Impedance spectroscopy (IS) is a method that provides critical insights into the electrical conductivity of materials. It allows for the identification of the types of charge carriers, whether electronic or ionic. This technique is also instrumental in assessing the suitability of nanocomposite films for use in electrochemical energy devices and battery applications.

**Fig. 7:**
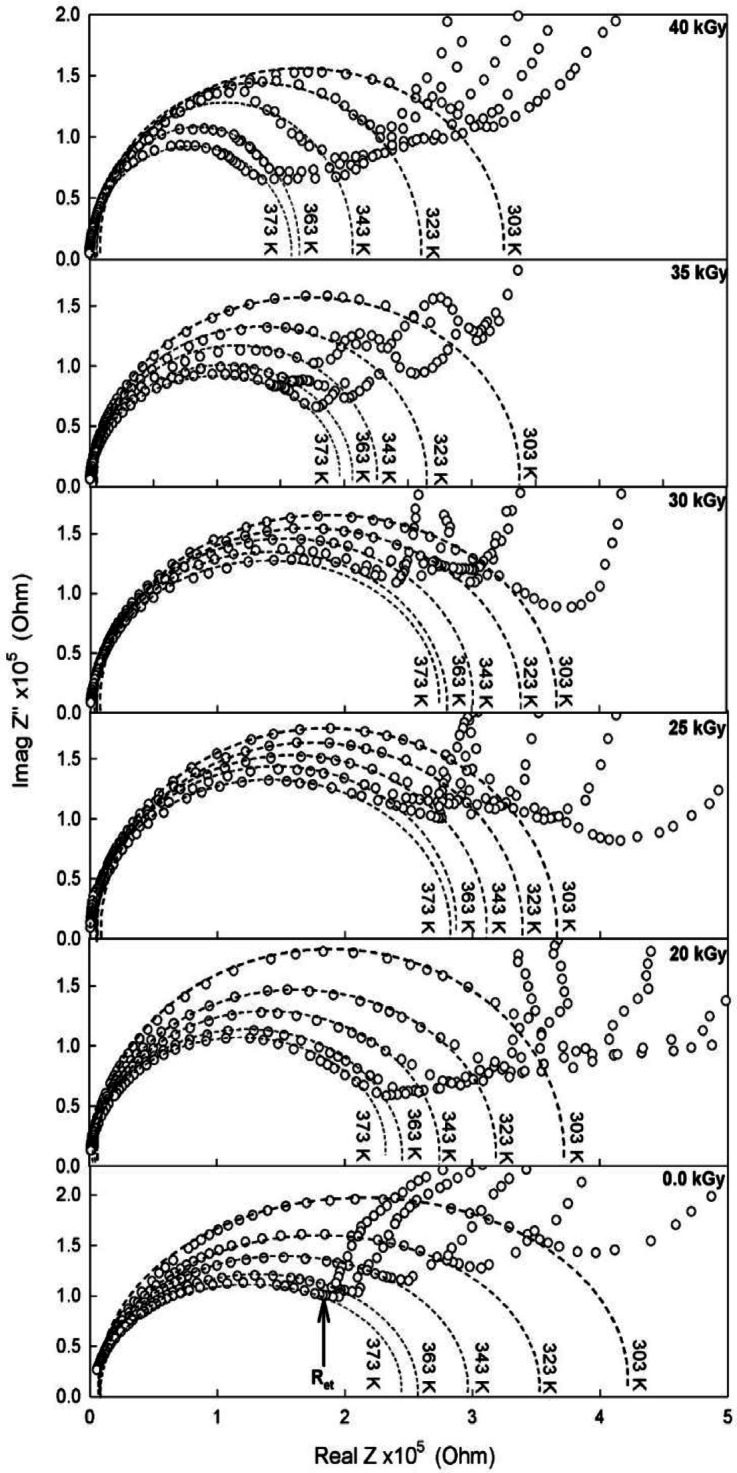
Nyquist plot of the studied nanocomposite polymer films recorded in the temperature range 303–373 K and under different irradiation doses.

The impedance spectroscopy spectrum for the studied PVA/PVP/Zn^2+^ films, are displayed in Fig. 7. The Nyquist plot in Fig. 7 illustrates the complex plane, depicting the relationship between *Z’* (real part of impedance) and *Z’’*(imaginary part of impedance). This relationship can be mathematically interpreted using an equivalent electrical circuit model, which represents the physico-electrical interaction between the electrode and electrolyte. These circuit models are constructed using ideal components such as resistors (R), capacitors (C), and inductors (L). By configuring these components in series or parallel, it is possible to accurately signify the impedance spectroscopy spectra of a two or three-electrode system. In an electrochemical system, impedance parameters are largely influenced by several key factors, including ion diffusion, electrochemical double layer formed, ion-adsorption mechanisms, electrical resistance, conductivity of the nanocomposite films, and electrochemical reactions. The impedance behavior of a material or system can often be modeled using a simple resistor (R) and capacitor (C) circuit. The impedance Z of this RC circuit depends on the values of R and C which can be expressed as^55^.

11 $$\:{Z}^{*}={R}_{s}+\frac{1}{i\omega\:C}$$

The impedance spectra of materials show a complex behaviour that cannot be fully captured by the simple RC equivalent circuit in Eq. (11). This complexity suggests the involvement of multiple interacting processes within the material, necessitating a more nuanced equivalent circuit model. A Randles circuit^56^ provides a more suitable model for analysing such spectra. The impedance of the Randles circuit is given by^56^:

12 $$\:{Z}^{*}={R}_{s}+\frac{1}{1+\left(i\omega\:{C}_{dl}{R}_{et}\right)}+{Z}_{W}$$

In this model, a series resistor *R*_*s*_ represents the solution or bulk resistance. A parallel capacitor *C*_dl_ accounts for the double-layer capacitance, while a parallel charge transfer resistance R_et_ reflects the resistance due to faradaic processes, such as charge transfer at the electrode interface. Additionally, a Warburg impedance element *Z*_W_^57^ is included in series to represent diffusional effects, which are especially significant in electrochemical systems.

The Randles circuit has been employed for decades by researchers to model systems involving charge transfer at the electrode-electrolyte interface. This equivalent circuit allows for the correlation between the frequencies associated with the solution resistance (*R*_s_) and the capacitance of the double layer (*C*_dl_​). From the Nyquist plot (Fig. 7), it can be inferred that the semicircle arises from *C*_dl_​, indicating a single time constant. The diameter of this semicircle corresponds to the polarization resistance, while the solution resistance is obtained from the high-frequency intercept on the real axis^56^.

The Nyquist curves in this figure display two distinct regions. The first region, occurring at high frequencies, features a semicircular arc resulting from the charge transfer resistance (*R*_*et*_​) combined in parallel with the double-layer capacitance (*C*_*dl*_​). The second region, observed as a linear spike at low frequencies, represents the Warburg resistance (Z_w_​​), which reflects the effect of blocking electrodes during ion migration^57^. The Warburg resistance phenomenon originates from the enhanced electric double-layer capacitance, which results from the accumulation of free charge carriers at the interfacial regions between the PVA/PVP-based electrolyte and the electrodes^56,57^. The Warburg resistance (Z_w_​) is associated with the charge transfer process and typically appears as a spike in the low-frequency region of the Nyquist plot, which is characteristic of ion diffusion processes^55,56^. The presence of a depressed semicircle suggests that ion relaxation follows a non-Debye behavior, indicating a distribution of relaxation times. Additionally, the low-frequency spike reflects the contribution of the electric double-layer capacitance at the electrode/electrolyte interface^57^. Furthermore, Warburg impedance is associated with diffusion processes of ions within the electrolyte and at the electrode surface. It reflects the time-dependent response of the system, particularly during the charging and discharging phases.

Furthermore, Fig. 7 shows that with increasing temperature, the semi-circular portion of the spectrum progressively reduces, becoming particularly diminished at 373 K. This trend implies that the resistive component in the composite film becomes more prominent than the capacitive one, likely due to the random orientation of dipoles induced within the chains connected to the main backbone. Furthermore, this behavior suggests that conduction within the sample is primarily driven by the movement of induced ions.

### Dielectric and electric modulus spectra

**Fig. 8:**
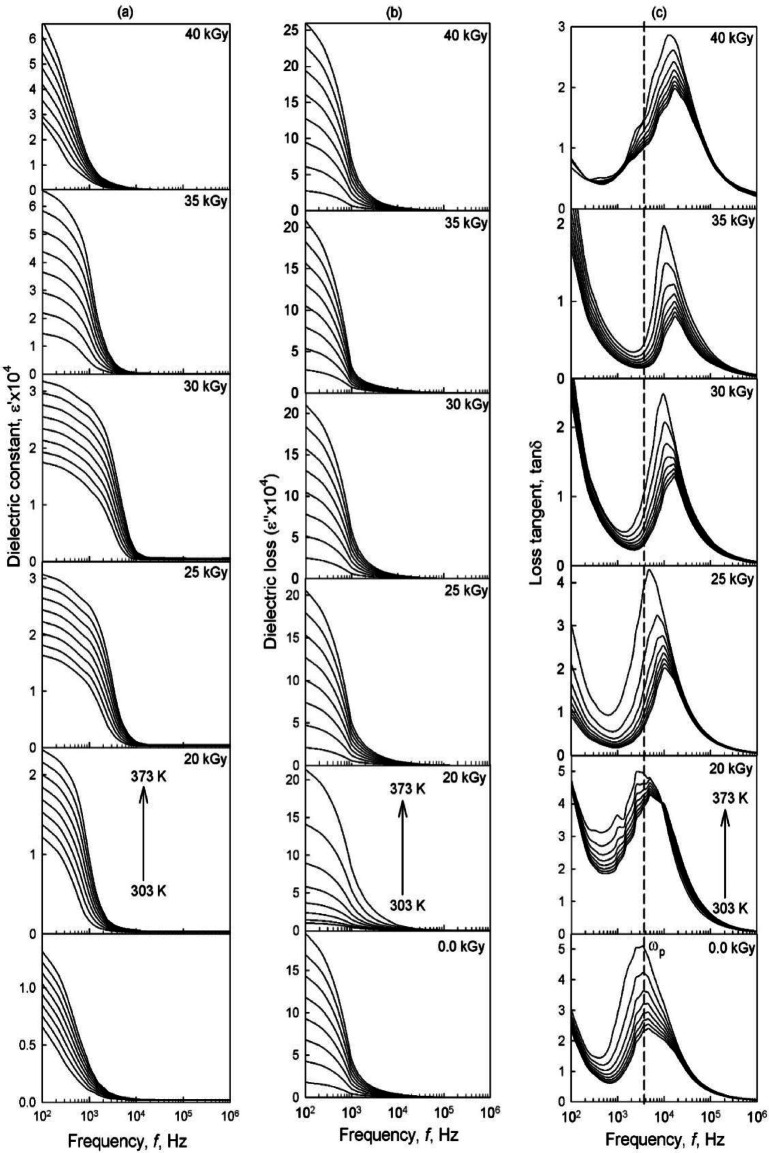
Dependence of the real part (ε′), imaginary part (ε″), and loss tangent (tan δ) as a function of frequency for the investigated gel electrolyte polymer nanocomposite films in the temperature range of 303–373 K and at different irradiation doses.

Dielectric measurements are crucial for understanding polymer systems’ ion-polymer interactions and conduction mechanisms. For PVA/PVP/Zn^2+^ nanoparticle films, dielectric properties such as dielectric constant (ε′), dielectric loss (ε′′), were evaluated with an impedance analyzer across a frequency range of 0.1 kHz to 1.0 MHz and temperatures between 303 and 373 K, as illustrated in Fig. 8a,b. These properties reveal changes in the electrical behavior of the nanocomposite films with temperature and frequency, reflecting ion dynamics, polarization, and energy dissipation within the polymer structure. Both ε′ and ε′′ display similar trends: high dielectric constant values are observed at low frequencies, primarily due to Maxwell–Wagner ionic conduction and electrode polarization, where ions accumulate at the electrode-electrolyte interface^57^. With increasing frequency, the dielectric constant decreases and eventually stabilizes as the electric field oscillations hinder ion diffusion in the field direction, keeping most ions within the bulk of the material and reducing the dielectric constant with higher frequencies^55^. This behavior suggests that the PVA/PVP/Zn^2+^ polymer blend exhibits non-Debye characteristics, with frequency-dependent ion diffusion and space charge regions^55,57^.

The dielectric constant also rises with irradiation doses, which can be attributed to the increased density of charge carriers in the space charge accumulation region, enhancing the equivalent capacitance. The dielectric constant increases at low frequencies with rising temperatures, indicating improved ion mobility within the polymer matrix^55^. This increase is further influenced by the PVA/PVP matrix’s amorphous and crystalline regions, which provide hopping sites for charge carriers. As temperature rises, the crystalline regions of the polymer progressively dissolve into the amorphous phase, further facilitating ion mobility.

The variation of the loss tangent (tan δ) of the (PVA/PVP)/Zn^2+^ films with frequency in the temperature range of 303–373 K is illustrated in Fig. 8c. A careful analysis of this variation provides valuable insights into the dielectric relaxation processes occurring in the system. The loss tangent is governed by the ratio of dissipated energy (ε′′) to stored energy (ε′) in the sample. This figure is marked by distinct relaxation peaks, which are attributed to dipolar relaxation processes arising from ion-dipole complexation within the polymer segments. The asymmetrical nature of these peaks confirms the semi crystalline structure of the (PVA/PVP)-based composite^55^. To further interpret the relaxation dynamics, the figure has been divided into three regions: first region at low-frequency region (< 10³ Hz): This region is characterized by the α-relaxation process, which is associated with the rotational motion of lateral functional groups around the central chain axis of the polymer matrix. This process dominates in the low-frequency range^58^. Second region lie in the moderate-frequency (< 10^5^): This region shows a relaxation peak around 30 kHz, corresponding to the β-relaxation process. This peak is related to the crankshaft-like motion of polymer segments, involving only a few segments. Polar functional groups such as N-O, C = O, O-H, and C-O, attached laterally to the polymer backbone, contribute to this β-relaxation^58^. Third region in the high-frequency (> 10^5^): this region is attributed to the γ-relaxation process, which involves dipolar relaxation due to the movement of induced charge carriers (ions/electrons) along with polymer segmental motion^59^. Furthermore, at lower frequencies, the increase in tan δ is due to the dominance of ohmic elements over the capacitive behavior of the composite. As the frequency rises, tan δ reaches a peak and then gradually decreases, reflecting the growing influence of capacitive components in the sample^58,59^. Additionally, a notable shift of the peak towards higher frequencies is observed with increasing irradiation doses, indicating that the relaxation time (τ = 1/ω_p_) decreases. The incorporation of conductive Zn ions into the dielectric PVA/PVP blend leads to interfacial polarization, forming new interfacial regions between the polymer blend and the filler. As Zn ions accumulate in these interfacial regions, the peaks shift to higher frequencies, reducing the relaxation time (τ). The relaxation times for the studied films are 2.5 × 10^−4^, 1.4 × 10^−4^, 1.1 × 10^−4^, 0.92 × 10^−4^, and 0.8 × 10^− 4^ s for doses 0, 20, 25, 30, 35, and 40 kGy in sequence. The best performance was observed for the sample irradiated with 40 kGy, which showed excellent compatibility between the filler and the PVA/PVP blend. This sample also achieved the greatest enhancement in the amorphous regions, as confirmed by XRD and FT-IR analyses, making it highly suitable for supercapacitor applications.

#### Electric modulus formalism

**Fig. 9:**
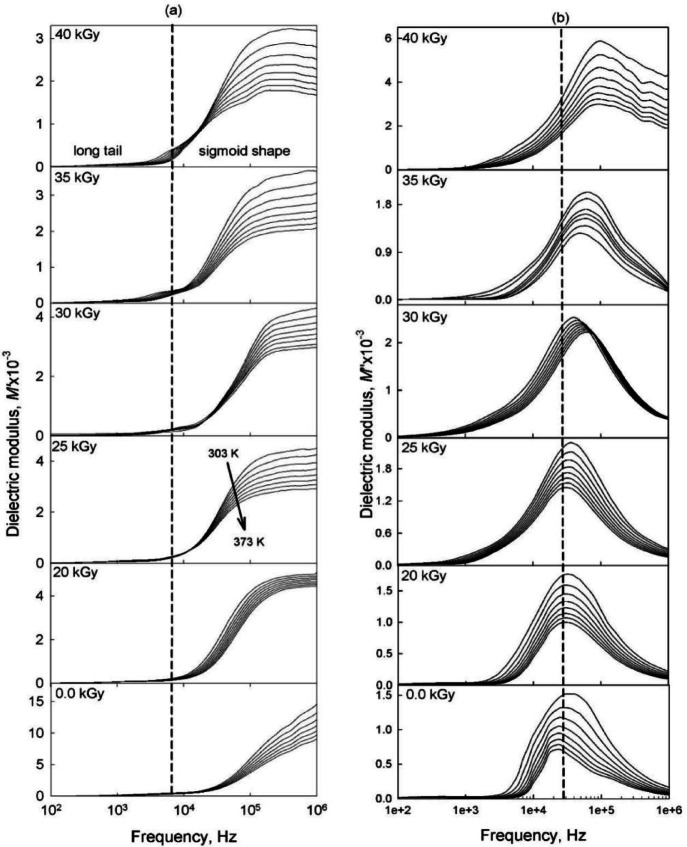
Dependence of the real electric modulus (*M*′), and imaginary part (*M*″), as a function of frequency for the investigated gel electrolyte polymer nanocomposite films in the temperature range of 303–373 K and at different irradiation doses.

The electric modulus formalism is effective in identifying the dominant conduction mechanism in polymer electrolytes and in mitigating the effects of electrode polarization. Figure 9a illustrates the variation of *M'* values with frequency for PVA/PVP electrolyte blends doped with different irradiation doses at room temperature. The spectra consist of two parts: a long tail at lower frequencies and a sigmoid-shaped region at higher frequencies. The long tail suggests the complete suppression of electrode polarization and indicates the capacitive nature of the PVA/PVP composite films^55^. The observed decrease in *M'* values with increasing irradiation dose suggests that ion movement occurs through hopping, confirming the dominant conduction mechanism in these composite samples^24^. Figure 9b shows the frequency dependence of *M''* values at different temperatures for the complex films. The *M'' *curves display a clear loss peak, attributed to the migration of induced charge carriers (Zn, O, S, and H ions), suggesting relaxation of the host matrix’s main chain^60^. The shift of the characteristic relaxation peak toward higher frequencies indicates a decrease in relaxation time, attributed to the formation of new interfacial regions with increasing irradiation, which facilitates the movement of induced charge carriers and confirms that the dominant conduction mechanism is of the hopping type^60^.

## Conclusion

Zn^2+^-doped PVA/PVP nanocomposite films, prepared via in situ methods and exposed to electron beam irradiation, exhibited significant improvements in structural, thermal, and dielectric properties. XRD and FTIR analyses confirmed the successful incorporation of ZnO and ZnS nanoparticles into the polymer matrix, with irradiation enhancing crystallinity and increasing the fraction of free ions. FESEM images showed uniform dispersion of zinc nanoparticles within the porous PVA/PVP network. Thermal analysis (DSC and TGA) revealed multistage decomposition and improved thermal stability, reflected in an increase of glass transition temperature (*T*_g_) from ~ 33.2 °C to 35.6 °C, higher melting points, and increased activation energy. Dielectric studies indicated enhanced ionic conductivity, evidenced by a reduction in Nyquist semicircle diameters with rising temperature. These results demonstrate that the PVA/PVP/Zn^2+^ nanocomposite films are promising candidates for advanced applications, particularly in supercapacitor devices, owing to their superior thermal, structural, and electrical performance.

## Data Availability

The datasets used and/or analyzed during the current study are available from the corresponding author on reasonable request.
